# Preexposure Prophylaxis for Prevention of HIV Acquisition Among Adolescents: Clinical Considerations, 2020

**DOI:** 10.15585/mmwr.rr6903a1

**Published:** 2020-04-24

**Authors:** Mary R. Tanner, Peter Miele, Wendy Carter, Sheila Salvant Valentine, Richard Dunville, Bill G. Kapogiannis, Dawn K. Smith

**Affiliations:** ^1^Division of HIV/AIDS Prevention, National Center for HIV/AIDS, Viral Hepatitis, STD, and TB Prevention, CDC; ^2^Division of Antiviral Products, Center for Drug Evaluation and Research, U.S. Food and Drug Administration, Washington, DC; ^3^Division of Adolescent and School Health, National Center for HIV/AIDS, Viral Hepatitis, STD, and TB Prevention, CDC; ^4^Maternal and Pediatric Infectious Disease Branch, Eunice Kennedy Shriver National Institute of Child Health and Human Development, Bethesda, Maryland

## Abstract

Preexposure prophylaxis (PrEP) with antiretroviral medication has been proven effective in reducing the risk for acquiring human immunodeficiency virus (HIV). The fixed-dose combination tablet of tenofovir disoproxil fumarate (TDF)/emtricitabine (FTC) was approved by the U.S. Food and Drug Administration (FDA) for use as PrEP for adults in 2012. Since then, recognition has been increasing that adolescents at risk for acquiring HIV can benefit from PrEP. In 2018, FDA approved revised labeling for TDF/FTC that expanded the indication for PrEP to include adolescents weighing at least 77 lb (35 kg) who are at risk for acquiring HIV. In 2019, FDA approved the combination product tenofovir alafenamide (TAF)/FTC as PrEP for adolescents and adults weighing at least 77 lb (35 kg), excluding those at risk for acquiring HIV through receptive vaginal sex. This exclusion is due to the lack of clinical data regarding the efficacy of TAF/FTC in cisgender women.

Clinical providers who evaluate adolescents for PrEP use must consider certain topics that are unique to the adolescent population. Important considerations related to adolescents include PrEP safety data, legal issues about consent for clinical care and confidentiality, the therapeutic partnership with adolescents and their parents or guardians, the approach to the adolescent patient’s clinical visit, and medication initiation, adherence, and persistence during adolescence. Overall, data support the safety of PrEP for adolescents. PrEP providers should be familiar with the statutes and regulations about the provision of health care to minors in their states. Providers should partner with the adolescent patient for PrEP decisions, recognizing the adolescent’s autonomy to the extent allowable by law and including parents in the conversation about PrEP when it is safe and reasonable to do so. A comprehensive approach to adolescent health is recommended, including considering PrEP as one possible component of providing medical care to adolescents who inject drugs or engage in sexual behaviors that place them at risk for acquiring HIV. PrEP adherence declined over time in the studies evaluating PrEP among adolescents, a trend that also has been observed among adult patients. Clinicians should implement strategies to address medication adherence as a routine part of prescribing PrEP; more frequent clinical follow-up is one possible approach.

PrEP is an effective HIV prevention tool for protecting adolescents at risk for HIV acquisition. For providers, unique considerations that are part of providing PrEP to adolescents include the possible need for more frequent, supportive interactions to promote medication adherence. Recommendations for PrEP medical management and additional resources for providers are available in the U.S. Public Health Service clinical practice guideline *Preexposure Prophylaxis for the Prevention of HIV Infection in the United States — 2017 Update* and the clinical providers’ supplement *Preexposure Prophylaxis for the Prevention of HIV Infection in the United States — 2017 Update: Clinical Providers’ Supplement* (https://www.cdc.gov/hiv/clinicians/prevention/prep.html).

## Introduction

Preexposure prophylaxis (PrEP) reduces the risk for acquiring human immunodeficiency virus (HIV) among persons at risk for HIV acquisition (*1*). In 2012 the U.S. Food and Drug Administration (FDA) approved the combination antiretroviral medication tenofovir disoproxil fumarate (TDF)/emtricitabine (FTC) for PrEP in adults. In 2018, FDA approved expanded labeling for TDF/FTC for use as PrEP to reduce the risk for sexually acquired HIV among adolescents weighing at least 77 lb (35 kg) (*2*). In 2019, FDA approved the combination product tenofovir alafenamide (TAF)/FTC as PrEP for adolescents and adults weighting at least 77 lb (35 kg), excluding those at risk for acquiring HIV through receptive vaginal sex due to the lack of clinical data regarding the efficacy of TAF/FTC in cisgender women (*3*).

The expansion of PrEP labeling to include adolescents is an important development because some adolescents in the United States remain at risk for acquiring HIV. In 2018, HIV diagnoses among persons aged 15–19 years totaled 1,688 (rate: 8.0 per 100,000 persons) (*4*). Nationally, adolescents have lower HIV diagnosis rates than persons in their 20s, who had the highest rates of new HIV diagnoses in 2018 (*4*). However, a study of young gay, bisexual, and other men who have sex with men (MSM) in Chicago found that the HIV incidence per 100 person-years for MSM aged 16–17 years was not significantly different than that among MSM aged 18–20 years (*5*). These data indicate that among certain populations, adolescents’ risk for HIV acquisition might be similar to that of adults, suggesting the need to enhance prevention efforts for persons aged <18 years. In addition, a modeling study using a network model and race-specific data from clinical trials has estimated that PrEP could meaningfully reduce HIV incidence among black and white adolescent sexual minority males (*6*). The number needed to treat to prevent an infection, described as a measure of PrEP’s efficiency, was similar for black adolescent sexual minority males in this study and adult MSM in a similar study (*6*,*7*).

HIV prevention strategies, including PrEP, are important for adolescents at risk for HIV acquisition, both for their well-being and that of their communities. Preventing HIV among adolescents is particularly important because adolescents with HIV might be at higher risk for inadequate medication adherence, failure to achieve viral suppression, viral rebound, and loss to follow-up than adults with HIV (*8*–*12*). These factors are associated with increased risks for development of antiretroviral resistance, impaired immune system function, and transmission of HIV to others (*13*–*17*). In addition, HIV acquisition earlier in life results in increased cumulative exposure to continuous, lifelong antiretroviral therapy (*18*).

Clinicians caring for adolescents should be aware of special considerations relevant to providing PrEP for this population. Current evidence indicates that many adolescents are not aware of PrEP, and many do not know how they could access PrEP if needed (*19*). In addition, certain providers who feel that they lack knowledge about providing PrEP to young persons might be unwilling to provide PrEP to adolescents and young adults (*20*).

Adolescence is a unique developmental stage, which is particularly germane to medication adherence. Overall, existing data for adults indicate that PrEP efficacy is a function of adherence (*21*). Compared with adults, adolescents are at increased risk for poor adherence to medications (*8*). Some factors that place adolescents at risk for poor medication adherence include biologic and behavioral co-occurring transitions (e.g., increasing autonomy, increasing importance of peers, and corresponding vulnerability to peer influence and stigma) and emerging but undeveloped cognitive capacity for organization and long-term planning (*22*,*23*). Clinical providers evaluating adolescents for PrEP use must be aware of the developmental changes occurring during adolescence and the additional considerations associated with providing PrEP in this context (*24*). Despite these challenges, clinical trials and real-world medical experience have determined that adolescents can successfully take PrEP (*25*–*27*). A recent study of MSM and transgender women aged 16–29 years found that at least three fourths of current PrEP users reported being ≥90% adherent to PrEP (*28*). Clinicians should be prepared to address and support medication adherence as a routine part of prescribing PrEP.

This report provides an overview of key topics about providing PrEP to adolescents on the basis of expert opinion and published literature. The topics covered in this review include PrEP safety data related to adolescents, legal issues about consent for clinical care and confidentiality, the therapeutic partnership with adolescents and their parents or guardians, approach to the adolescent patient’s clinical visit, and medication initiation, adherence, and persistence (Box 1). This report is intended for use by clinical providers who treat adolescent patients and for other health professionals and public health officials who provide referrals and supportive services related to adolescent health. The U.S. Public Health Service clinical practice guideline *Preexposure Prophylaxis for the Prevention of HIV Infection in the United States — 2017* and clinical providers’ supplement *Preexposure Prophylaxis for the Prevention of HIV Infection in the United States — 2017 Update: Clinical Providers’ Supplement* (https://www.cdc.gov/hiv/clinicians/prevention/prep.html) include recommendations for providing PrEP to adolescents (*1*).

BOX 1 **Key clinical considerations when prescribing HIV preexposure prophylaxis to adolescents**

**Medical management**

- Refer to the U.S. Public Health Service clinical practice guideline Preexposure Prophylaxis for the Prevention of HIV Infection in the United States (https://www.cdc.gov/hiv/clinicians/prevention/prep.html).

**PrEP safety**

- Overall, the available data support the safety of PrEP in adolescents.
- Clinical and laboratory-based monitoring is recommended for persons prescribed PrEP (https://www.cdc.gov/hiv/clinicians/prevention/prep.html).
- Additional research is needed to assess potential clinical impacts of TDF-related effects on bone density in adolescents.

**Legal issues**

- As of a broad legal review in 2017, no states specifically prohibit minors’ autonomous consent to PrEP.
- All states have statutes, regulations, or both that explicitly allow certain minors to consent to STI diagnosis and treatment.
- Allowing minors to autonomously consent to PrEP does not necessarily mean that care will remain confidential. Potential limitations to confidentiality include billing documentation, mandated reporting laws for child abuse and neglect, and others depending on the state.
- PrEP providers have to be familiar with the statutes and regulations regarding the provision of health care to minors in their jurisdictions.

**Therapeutic partnership**

- Adequate time for adolescents and their providers to discuss sensitive topics without parents or guardians present, also called time alone, is recommended beginning at age 11 years.
- PrEP providers must recognize adolescents’ autonomy to the extent allowable by law and include parents or guardians in the conversation about PrEP when it is safe and reasonable to do so.

**Clinical visits for preventive services**

- Obtaining a comprehensive history, including social and sexual history, is important to identifying adolescents’ needs. PrEP is only one of a number of interventions that might be indicated to address an adolescent’s health concerns.
- One approach to the history is the HEEADSSS (or HE2ADS3) format: asking adolescents about home, education or employment, eating, activities (peer related), drugs, sexuality, suicide or depression, and safety.
- One approach to the sexual history is the five Ps method: asking adolescents about partners, practices, protection from STIs, past history of STIs, and prevention of pregnancy.
- All adolescents need to receive developmentally appropriate sexual health education.

**Medication initiation, adherence, and persistence**

- Providers and clinical support staff can help adolescents prescribed PrEP by screening for barriers and providing pertinent guidance and support.
- Resources to overcome barriers related to medication and health care costs are available (https://www.cdc.gov/hiv/risk/prep/index.html).
- Adequate medication adherence is required for PrEP to prevent HIV acquisition. In PrEP clinical trials among adolescents, initial monthly clinic visits were associated with higher adherence than the later quarterly visits.
- Adolescents prescribed PrEP might benefit from more frequent, supportive interactions.
- Studies evaluating technology-focused interventions and other strategies to enhance adherence are ongoing.
- Regular screening for the need and desire to continue with PrEP is part of the ongoing care of adolescents prescribed PrEP. For adolescents who plan to continue taking PrEP, screening for barriers (e.g., transportation problems and insurance changes) and providing guidance and support for overcoming barriers can help them persist with PrEP.

**Abbreviations:** HIV = human immunodeficiency virus; PrEP = preexposure prophylaxis; STI = sexually transmitted infection; TDF = tenofovir disoproxil fumarate.

## Methods

This report was developed through collaboration between CDC subject matter experts and experts from other federal agencies. Input into this report came from subject matter expert recommendations, peer-reviewed literature, published guidelines, and a legal research database. The authors reviewed relevant peer-reviewed literature by conducting iterative searches in PubMed, beginning with broad search terms and then focusing on specific topics as guided by review of pertinent publications. English-language articles were reviewed without regard to date of publication if they addressed relevant topics and were focused on care of adolescents in the United States.

Published guidelines from other expert groups related to the medical care of adolescents, including the American Academy of Pediatrics, the Society for Adolescent Health and Medicine, and the U.S. Preventive Services Task Force, were reviewed. PubMed and ClinicalTrials.gov were used to identify sources relevant to adherence among participants in randomized PrEP trials, including interventions enrolling adolescent participants, and strategies to monitor and support adherence among adolescents. A review of legal issues related to provision of PrEP to adolescents was conducted during August–December 2017.

An online legal research service (Westlaw next) was searched using broad terms (“consent” and “minors”) to find any statute or regulation in all 50 states and the District of Colombia that addressed minors’ consent to health care services and diagnosis, treatment, or prevention of HIV, sexually transmitted infections (STIs), or both. A panel of assessors was created, consisting of an attorney and manuscript co-author and two epidemiologists. Each law was assessed using a questionnaire that addressed topics such as the age of majority, types of family planning services a minor may receive, and exceptions to the age of majority to consent to health care services (Supplementary Material, https://stacks.cdc.gov/view/cdc/86247). Results were then summarized to describe legal issues related to PrEP services for adolescents in each state and the District of Columbia.

## Prescribing Considerations for HIV PrEP Among Adolescents

### PrEP Safety Data

To effectively counsel patients, providers must be familiar with the safety data related to PrEP use among adolescents. Long-term clinical experience with the fixed-dose combination of TDF and FTC has shown the drug to be safe and well tolerated across different indications and age groups. The most common adverse reactions observed in adult PrEP trials include headache, abdominal pain, and decreased weight (*29*). The initiation of TDF/FTC for PrEP has been associated with self-limited start-up syndrome in approximately 10% of adults, consisting predominantly of mild to moderate gastrointestinal symptoms, headache, and fatigue; unintentional weight loss has also been reported (*30*–*32*).

Potential toxicities associated with use of TDF include effects on renal and skeletal systems. In adult PrEP trials, the risk for renal impairment with TDF/FTC was not significant compared with placebo, and the serum creatinine abnormalities that were observed were reversible when the medication was stopped (*21*). Similarly, although TDF is known to decrease bone mass when used for HIV treatment or prevention, the clinical significance is uncertain. In adult PrEP trials, the decreases in bone mineral density (BMD) observed while taking PrEP reversed when the medication was stopped (*33*,*34*).

Limited safety data from trials of TDF/FTC among HIV-uninfected adolescents further assist in the risk-benefit assessment of PrEP among adolescents. Two open-label trials of TDF/FTC for PrEP among adolescents have been conducted: 1) the Adolescent Trials Network 113 (ATN 113) study among persons aged 15–17 years in the United States (*26*) and 2) the Choices for Adolescent Methods of Prevention in South Africa (CHAMPS) PlusPills study among persons aged 15–19 years in South Africa (*25*). Both studies found that TDF/FTC for PrEP was safe and well tolerated among different adolescent populations at risk for HIV acquisition (*25*,*26*).

Published data from ATN 113 indicate that no confirmed laboratory abnormalities were considered to be related to TDF/FTC (*26*). One participant permanently discontinued the study drug because of grade 3 weight loss, which was assessed and determined to be possibly related to TDF/FTC; unintentional weight loss was the only grade 3 or 4 adverse event reported in more than one participant. No renal adverse events, elevations in serum creatinine levels, or bone fractures were reported in this study. Dual-energy x-ray absorptiometry (DXA) tests, used to assess BMD, indicated substantial increases from baseline BMD in the spine, hip, and total body at 48 weeks, as would be expected in growing adolescents. Although BMD Z-scores in the hip and spine did not change from baseline to 48 weeks, the total body Z-score decreased (-0.20; interquartile range: -0.3 to 0.0; p<0.001). Interpretation of these safety results might be affected by the overall low adherence to the study drug observed in ATN 113, particularly after week 12.

ATN 113 (*26*) and ATN 110, which studied PrEP among persons aged 18–22 years (*35*), had a preplanned extension phase for participants who had signs of bone or renal toxicity at 48 weeks while taking TDF/FTC. Participants in the extension phase were followed for 48 weeks after TDF/FTC discontinuation. For participants aged 15–19 years, spine and whole body BMD Z-scores remained below baseline after 48 weeks with no TDF/FTC; hip Z-scores recovered to baseline (*36*). ATN 117, a substudy of ATN 110 and 113, evaluated the association of serum 25-hydroxy vitamin D insufficiency and TDF exposure with bone toxicity, as defined by age-specific DXA criteria. The study demonstrated a gradient of bone toxicity risk, from lowest to highest of 1) vitamin D replete with poor TDF exposure, 2) vitamin D replete with high TDF exposure, 3) vitamin D insufficient with low TDF exposure, and 4) vitamin D insufficient with high TDF exposure. The study also reported a higher rate of bone toxicity in black persons, independent of vitamin D or TDF levels (*37*). The clinical impact of these findings is not known. A meta-analysis of 13 randomized PrEP trials indicated no increase in bone fractures among persons taking TDF-containing PrEP; however, the follow-up periods for included studies were variable, ranging from 4 months to 5 years, and might have been inadequate to fully evaluate long-term effects (*38*).

In addition to data from the open-label trials of TDF/FTC among uninfected adolescents, clinical experience with use of this medication for treatment of children and adolescents with HIV is substantial. The extrapolation of safety data from trials of TDF and FTC in adolescents with HIV is permissible because acquiring HIV does not affect the pharmacokinetics of TDF or FTC and the dosage for adolescents weighing at least 77 lb (35 kg) is the same whether used for treatment or prevention (i.e., 200 mg/300 mg once daily) (*29*). In treatment trials, TDF and FTC were well tolerated in adolescents with HIV (*39*,*40*). Adverse events of skin hyperpigmentation and anemia, which are described as common adverse drug reactions for pediatric patients in FTC and TDF/FTC labeling (*41*), were not reported among adolescents. The incidence of discontinuation due to adverse events was consistently low across all TDF and FTC trials with no specific treatment-limiting adverse effects or laboratory abnormalities identified. The renal and bone safety profiles among adolescents with HIV receiving TDF through 48 weeks were consistent with those among adults with and without HIV. No adverse events of Fanconi syndrome or tubulopathy were reported. All reported fractures were trauma related. Increases from baseline in spine and total body BMD were seen at weeks 24 and 48, although the mean rate of BMD gain at week 48 was less with TDF compared with placebo. Skeletal growth (i.e., height) appeared unaffected.

Clinical trials to evaluate TAF/FTC safety, efficacy, and adherence in adolescents have not been performed. Efficacy and safety data to support the FDA approval of TAF/FTC for PrEP were extrapolated from the DISCOVER trial in adult MSM and transgender women and from trials of TAF/FTC in adolescents with HIV (*42*).

Overall, the available data support the safety of PrEP in adolescents. As outlined in the U.S. Public Health Service clinical practice guideline, ongoing clinical and laboratory-based monitoring (e.g., HIV testing, creatinine monitoring, and STI screening) is recommended for persons prescribed PrEP (*1*). Additional research is needed to assess potential clinical impacts of TDF-related effects on bone density in adolescents. Evidence is insufficient to inform specific recommendations about vitamin D levels or vitamin D supplementation for all adolescents starting PrEP. The U.S. Public Health Service 2017 guidelines do not recommend routine use of DXA tests for persons taking PrEP; however, persons with a history of pathologic fracture or other bone health risk factors should be referred for consultation and management (*1*).

Familiarity with the available safety data allows for effective counseling to adolescents and, when appropriate, their parents or guardians. This knowledge will allow PrEP providers to effectively weigh benefits and risks, address safety-related questions, and explain the importance of the recommended evaluations for monitoring PrEP patients.

### Legal Issues

Legal issues about consent for clinical care, status as a legal minor, and confidentiality are important considerations for providing PrEP to adolescents. The legal framework varies considerably by state. In 2017, potentially applicable laws were reviewed to clarify issues of consent for clinical care and confidentiality about providing PrEP for adolescents (*43*). Minors’ legal ability to autonomously access PrEP was assessed on the basis of two elements in a state’s laws: 1) whether the state either explicitly included HIV infection in the law or designated HIV infection as an STI through another law and 2) whether the state explicitly included prevention in the law allowing minors to autonomously consent to care for STIs (*43*). Minors can still be legally allowed to autonomously consent to PrEP in certain states with laws lacking the two elements because other mechanisms, such as case law, might be used to categorize HIV infection as an STI, define treatment broadly to include prevention, or both.

The review determined that no states explicitly prohibit minors’ autonomous consent to PrEP (*43*). All states have statutes, regulations, or both that explicitly allow certain minors to consent to STI diagnosis and treatment (*43*). Seven states explicitly include HIV infection in the law allowing minors to autonomously consent to the service, or designate HIV infection as an STI through another law, and explicitly include prevention in the law allowing minors to autonomously consent to the services (*43*) (Table 1). Nine states broadly allow minors to autonomously consent to any health care services or procedures, arguably including PrEP; some states define minimum ages for minors consenting to health care services (*43*) (Table 2).

**TABLE 1:** States with laws that allow minors to autonomously consent to sexually transmitted infection care and explicitly include human immunodeficiency virus as a sexually transmitted infection — United States, 2017

State	Consent	HIV	Type of prevention
California	Allows minors to autonomously consent to preventive or prophylactic STI services Minors must be aged ≥12 years to autonomously consent to preventive services	Specifies that HIV infection is an STI through another law	Not specified
Colorado	Allows minors to autonomously consent to preventive or prophylactic STI services	Specifies that HIV infection is an STI through another law	Not specified
Delaware	Allows minors to autonomously consent to preventive or prophylactic STI services Minors must be aged ≥12 years to autonomously consent to preventive services	Specifies that HIV infection is an STI through another law	Not specified
Iowa	Allows minors to autonomously consent to preventive or prophylactic HIV services	Explicitly includes HIV infection prevention in law	Not specified
Montana	Allows minors to autonomously consent to preventive or prophylactic STI services	Specifies that HIV infection is an STI through another law	Not specified
North Carolina	Allows minors to autonomously consent to preventive or prophylactic STI services	Specifies that HIV infection is an STI through another law	Not specified
Oklahoma	Allows minors to autonomously consent to preventive or prophylactic STI services	Specifies that HIV infection is an STI through another law	Not specified

**Abbreviations:** HIV = human immunodeficiency virus; STI = sexually transmitted infection.

**TABLE 2:** States with laws that broadly allow minors to autonomously consent to any health care services or procedures — United States, 2017

State	Provision
Alabama	Minors must be aged ≥14 years to autonomously consent to health care services.
Alaska	No age restriction defined.
Arkansas	No age restriction defined.
Idaho	Minors must be aged ≥14 years to autonomously consent to health care services.
Kansas	Minors must be aged ≥16 years to autonomously consent to health care services.
Louisiana	No age restriction defined.
Mississippi	Minors must be aged ≥18 years to autonomously consent to health care services. (Age of majority is 21 years.)
Pennsylvania	Minors must be aged ≥18 years to autonomously consent to health care services. (Age of majority is 21 years.)
South Carolina	Minors must be aged ≥16 years to autonomously consent to health care services.

Allowing minors to autonomously consent to PrEP does not mean that access to the health care service will remain confidential. Providers are permitted to disclose a minor’s autonomous access to HIV and STI services to the minor’s parents or guardians in 23 states (*43*). In addition, billing documentation from commercial health insurance companies in an explanation of benefits provides a potential mechanism of disclosure of services provided to adolescents covered under their parents’ or guardians’ health insurance (*44*).

Title X–funded clinics provide comprehensive family planning and related preventive services and HIV testing, screening, and treatment for STIs (*45*). Furthermore, Title X–funded clinics cannot require parental consent and must maintain confidentiality (*46*). Whereas Title X clinics could provide a place for adolescents to access PrEP confidentially (*30*), clarification is needed regarding the U.S. Department of Health and Human Services’ policies on provision of PrEP under Title X funding (*47*). Funding restrictions might also limit widespread access to PrEP in Title X–funded clinics (*46*).

Even in states that allow minors to consent to certain aspects of their medical care, in certain circumstances a provider might need to breach an adolescent’s confidentiality. In several states, mandatory reporting laws require providers to report child abuse and neglect, including sexual abuse and sex trafficking (*48*).

PrEP providers have to be familiar with the statutes and regulations regarding the provision of health care to minors in their jurisdictions. State and local health departments might be able to serve as resources for questions about these topics.

### Therapeutic Partnership

Another unique challenge of providing PrEP to adolescents is how to best partner with both the adolescents and their parents or guardians, if applicable to the adolescent’s situation. Adolescence is characterized as a period of transition for youths as they mature into adulthood; likewise, the role of parents shifts during this time, with increasing autonomy given to the adolescent (*49*). Parental monitoring, which includes parental solicitation and adolescent disclosure of information as well as parental control, is associated with reduced risk behavior (*50*) and increased protective behavior, especially when parents recognize the autonomy of adolescents (*51*). Parental recognition of the autonomy of adolescents is associated with adherence to medications for chronic disease (*52*). In addition, parent-adolescent conversations about HIV have been associated with adolescent PrEP knowledge, illustrating that parents can be an important source of HIV prevention knowledge (*53*).

Health care providers can be important partners with adolescents and with their parents or guardians in promoting healthy behaviors through a triadic approach (child, parent, and provider), whether working directly with adolescents or with their parents or indirectly through supporting a healthy parent-child relationship (*54*,*55*). One study examining parent-adolescent dyads found a significant correlation between parents and adolescents with regard to their desire for sexual health information from providers, suggesting that when adolescent patients have sexual health questions for providers, their parents might have those same questions as well (*56*).

For health care providers, working with parents to support their child’s care, especially for sensitive health services such as PrEP, can conflict with protecting the adolescent’s confidentiality (*49*) and can be complicated to negotiate (*57*). Studies have indicated that adolescents are less likely to seek services if they are concerned about confidentiality, yet parents might want to remain informed of discussions between adolescents and their providers even when those discussions are considered confidential (*57*). However, adequate time for adolescents and their providers to discuss sensitive topics without parents present, also called time alone, is associated with adolescents receiving more sexual health services (*58*). Current American Academy of Pediatrics guidelines recommend that time alone begin at approximately age 11 years (*59*), a process parents can facilitate by promoting time alone gradually through teen years, as appropriate. Time alone can be especially important for adolescent MSM, who might be reluctant to share information with providers with their parents present (*60*). Similar concerns exist for adolescents who inject drugs because they might be hesitant to disclose information about drug use to providers in the presence of their parents. Overall, PrEP providers must partner with the adolescent patient for PrEP decisions, recognizing the adolescent’s autonomy to the extent allowable by law, and include parents in the conversation about PrEP when it is safe and appropriate to do so.

### Approach to the Clinical Visit for Prevention Services

A clinical approach tailored for the adolescent patient can enhance patient-provider communication about PrEP or other health-related topics. Obtaining a comprehensive history, including social and sexual history, is important to identifying adolescents’ health needs and providing individualized recommendations and counseling (*61*).

Providers can set the tone for the visit by introducing themselves to adolescents before the parents or guardians (*62*). If a parent or guardian is present, the adolescent patient should have the opportunity to have time alone with the provider. A helpful approach is to conduct the interview while the patient is still clothed and seated somewhere other than the examination table. The provider should inform the adolescent of the confidentiality of the provider-patient conversation, including the circumstances that might require confidentiality to be breached.

During the confidential interview with the adolescent patient, the provider might consider beginning with less sensitive topics. One well-described approach is the HEEADSSS (or HE^2^ADS^3^) format: asking the adolescent about home, education or employment, eating, activities (peer related), drugs, sexuality, suicide or depression, and safety (*62*,*63*). One approach to taking the adolescent’s sexual history is the five Ps method: asking the adolescent about partners, practices, protection from STIs, past history of STIs, and prevention of pregnancy (*64*). An important aspect of the sexual history includes screening for transactional sex to evaluate whether adolescents are engaging in sex in exchange for money, shelter, food, drugs, hormones (for transgender adolescents), or other resources. In all instances, the provider can use open-ended questions to facilitate effective communication. During certain parts of the history, including questions about substance abuse, the provider could begin the conversation by asking about activities of the adolescent’s friends (*62*). Active listening techniques (e.g., reflection, restatement, clarifying, and supportive statements) might encourage open discussion (*44*). Respectful, nonjudgmental language should be used, including avoiding assumptions about sexual orientation, gender identity, or the sex of the patient’s sex partners. Affirming the patient’s gender identity by using the preferred name and gender terms is also important. Training and resources are available to help providers gain skills in effective communication and provision of culturally competent care (*65*,*66*).

PrEP is one possible component in a comprehensive approach to health for adolescents who inject drugs or engage in sexual behaviors that place them at risk for acquiring HIV. All adolescents need to receive developmentally appropriate sexual health education (*67*). CDC’s STI treatment guidelines recommend that providers counsel adolescents about the sexual behaviors that are associated with risk for acquiring STIs and educate patients about evidence-based prevention strategies, including a discussion about abstinence and other risk-reduction behaviors (e.g., consistent and correct condom use and reduction in the number of sex partners) (*64*). The U.S. Preventive Services Task Force recommends intensive behavioral counseling for all sexually active adolescents. The recommendation suggests that this counseling can be conducted through one-on-one conversations, videos, written materials, or telephone support (*68*).

Certain youths might have serious challenges such as mental illness, substance use, juvenile justice system involvement, unstable housing, food insecurity, transactional sex, or other concerns. PrEP is only one of a number of interventions that might be indicated for an adolescent’s needs. Reports of abuse, neglect, or sex trafficking must be reported to the relevant authorities. Adolescents with evidence of mental health concerns might require referral to mental health resources. Adolescents who are using drugs illicitly need to receive clinical counseling and referral for services as indicated. Those who disclose homelessness or other needs can benefit from referral to social services or links to community-based resources.

### Medication Initiation, Adherence, and Persistence

The U.S. Public Health Service 2017 guidelines recommend that persons starting PrEP should initiate the medication within 7 days of a negative HIV test (*1*). However, barriers to obtaining PrEP, such as lack of insurance coverage, can delay initiation and might also adversely affect adherence and persistence for those who have started PrEP (*69*). Providers and clinical support staff can help adolescents prescribed PrEP by screening for barriers and providing pertinent guidance and support. Resources to overcome barriers related to medication costs are available, including the U.S. Department of Health and Human Services’ Ready, Set, PrEP program (*70*,*71*). Discussion of options to address additional costs of PrEP care (e.g., clinical visits and laboratory tests) are available from multiple sources, including CDC’s PrEP website (*72*,*73*).

Adequate medication adherence is required for PrEP to prevent HIV acquisition. Adherence was demonstrated to have a direct influence on PrEP efficacy in four randomized PrEP trials among adults. As summarized in a review, drug levels within the range reported to protect against HIV transmission were obtained in 50%–81% of participants, and post hoc analyses from these initial studies indicated that protection against HIV ranged from 92% to 100% among participants whose drug levels suggested they were taking the medication daily (*21*). These findings are in contrast with results of two PrEP studies that did not demonstrate PrEP efficacy. In both the VOICE (Vaginal and Oral Interventions to Control the Epidemic) and FEM-PrEP (Preexposure Prophylaxis Trial for HIV Prevention Among African Women) studies, no difference was reported in HIV incidence between PrEP and placebo groups (*74*,*75*). However, in the VOICE trial, tenofovir was detected in only 29%–30% of random plasma samples from participants randomized to receive oral tenofovir-containing PrEP (*74*). In the FEM-PrEP study plasma tenofovir levels were evaluated at specific timepoints, and among women randomized to receive PrEP, target drug levels were found in only 15%–26% of women who seroconverted during the study and 24%–37% of women who did not seroconvert (*75*). These data highlight the importance of the recommendation to carefully monitor and support adherence for all persons prescribed PrEP (*21*).

Because of the dynamic physical, cognitive, psychological, and social shifts occurring during adolescence, PrEP use patterns among U.S. adolescents and young adults enrolled in the ATN PrEP studies demonstrated challenges with achieving the high levels of adherence needed for efficacy (*76*,*77*). In these studies, quarterly clinic visits were associated with poorer medication adherence among both younger (*26*) and older youths than the initial monthly visits (*35*).

Despite the evidence demonstrating that adolescents commonly experience difficulties with adherence, efficacious adherence support interventions for clinical practice are limited. Youths with diagnosed HIV have benefited from youth-friendly mobile technologies, including cell phone support (*78*) and short message service (SMS) reminders (*79*). The interventions that have been developed specifically for PrEP include combinations of enhanced counseling, feedback of objective adherence measures, and SMS (*80*). The adherence challenges noted in the ATN PrEP studies occurred although youths received integrated next step counseling (iNSC), which is an interactive, client-centered motivational approach used to support adherence among participants in the Preexposure Prophylaxis Initiative (iPrEx) trial (*81*). This finding suggests that further interventions are needed to help adolescents adhere to PrEP. Real-time adherence feedback via dried blood spot tenofovir concentration measurements is the focus of several clinical trials under way to determine its utility in tailoring support to improve adherence outcomes (*82*,*83*). Urine tenofovir concentration as a possible point-of-care assay is also under study (*84*,*85*) (Box 2).

BOX 2 Clinical trials related to HIV preexposure prophylaxis adherence among adolescents*

**Table:** 

**Trial name**	**ClinicalTrails.gov URL**
Pre-exposure Prophylaxis (PrEP) Adherence Enhancement Guided by iTAB and Drug Levels for Women (AEGiS)	https://clinicaltrials.gov/ct2/show/NCT02584140
Evaluating the Pharmacokinetics, Feasibility, Acceptability, and Safety of Oral Pre-Exposure Prophylaxis for HIV Prevention During Pregnancy and Postpartum	https://clinicaltrials.gov/ct2/show/NCT03386578
Tenofovir Adherence to Rapidly Guide and Evaluate PrEP and HIV Therapy (TARGET)	https://clinicaltrials.gov/ct2/show/NCT03012607
Validation of a Urine Assay to Measure Tenofovir Levels in Patients Taking Tenofovir Alafenamide	https://clinicaltrials.gov/ct2/show/NCT03350672
LYNX: A Novel Mobile App to Support Linkage to HIV/STI Testing PrEP for Young Men Who Have Sex With Men	https://clinicaltrials.gov/ct2/show/NCT03177512
P3 (Prepared, Protected, emPowered) (P3)	https://clinicaltrials.gov/ct2/show/NCT03320512
Get Connected Efficacy Trial	https://clinicaltrials.gov/ct2/show/NCT03132415
Mobile-Based Application “MyChoices”	https://clinicaltrials.gov/ct2/show/NCT03179319
*As of September 2019.	

Although practice varies among clinicians providing PrEP to adolescents (*86*), the decline of adherence observed after reduction of clinic visit frequency in the ATN studies seems to suggest that in general, youths might benefit from more frequent, supportive interactions. This is a consideration in the revised TDF/FTC labeling (*29*) and is consistent with management guidelines for other clinical interventions affecting youth, such as pregnancy prevention (*87*). Other potentially useful clinical approaches might include more flexible clinic schedules (e.g., after-hours availability), peer navigators or adherence buddies, and other youth-friendly strategies. Although many experts deem such approaches helpful, further evaluation is needed to understand whether their use improves adherence to care and medication. ATN (*88*) has several ongoing clinical trials evaluating innovative technology-focused interventions and other strategies to improve use of HIV prevention services and PrEP among adolescents and young adults at risk for HIV acquisition (*89*–*92*) (Box 2).

In addition, sociodemographic and behavioral factors can influence medication adherence for adolescents prescribed PrEP (*93*). Screening for potential barriers to adherence (e.g., unstable housing) would allow providers to offer resources, referrals, or counseling when indicated. Adherence (i.e., taking PrEP medication as prescribed) is crucial to PrEP’s effectiveness. Persistence (i.e., continuing to take PrEP while the adolescent remains at risk for acquiring HIV) is another important factor. A study of young MSM (aged 16–29 years) reported that 33% of 197 males who reported using PrEP in the past 6 months had discontinued PrEP by the time of the study interview (*94*). The most commonly reported reasons for discontinuing PrEP included difficulty getting to appointments, lapse in insurance coverage, and perception of being no longer at risk for HIV (*94*). Regular screening for the need and desire to continue with PrEP is part of the ongoing care of an adolescent prescribed PrEP. For those who plan to continue taking PrEP, screening for barriers to persistence (e.g., transportation problems and insurance changes) and providing guidance and support for overcoming barriers can help adolescents continue taking PrEP. Circumstances, attitudes, and behaviors will change over time, and regular conversations about both adherence and persistence will promote patient-provider communication about these important factors.

## Conclusion

PrEP is a safe and effective intervention to reduce the risk for HIV acquisition. Adolescents should be screened for behaviors that put them at risk for acquiring HIV. In adolescents for whom PrEP is indicated, PrEP can be offered as part of a comprehensive approach tailored to their specific needs. In addition to the recommendations provided in the U.S. Public Health Service clinical practice guideline (*1*), providers prescribing PrEP for adolescents also should consider the relevant legal framework in their states; the importance of optimizing a therapeutic partnership with adolescents, and when possible, their parents or guardians; techniques to optimize adolescents’ clinical visits; and the importance of managing medication adherence. The data available from PrEP trials in adolescents suggest that adolescents might benefit from more frequent contact with clinical staff to support medication adherence. Resources are available to assist clinical providers with navigating issues related to prescribing PrEP. The Clinician Consultation Center offers a PrEP clinical consultation line for health care providers (855-448-7737) (*95*). The U.S. Public Health Service PrEP clinical practice guideline and clinician’s supplement (https://www.cdc.gov/hiv/clinicians/prevention/prep.html) provide guidance and references to other resources.
